# Body composition and functional limitation in COPD

**DOI:** 10.1186/1465-9921-8-7

**Published:** 2007-01-29

**Authors:** Mark D Eisner, Paul D Blanc, Steve Sidney, Edward H Yelin, Phenius V Lathon, Patricia P Katz, Irina Tolstykh, Lynn Ackerson, Carlos Iribarren

**Affiliations:** 1 Division of Occupational and Environmental Medicine and Division of Pulmonary and Critical Care Medicine, Department of Medicine, University of California, San Francisco, USA; 2 Division of Research, Kaiser Permanente, Oakland, CA, USA; 3 Institute for Health Policy Studies, Department of Medicine, University of California, San Francisco, USA

## Abstract

**Background:**

Low body mass index has been associated with increased mortality in severe COPD. The impact of body composition earlier in the disease remains unclear. We studied the impact of body composition on the risk of functional limitation in COPD.

**Methods:**

We used bioelectrical impedance to estimate body composition in a cohort of 355 younger adults with COPD who had a broad spectrum of severity.

**Results:**

Among women, a higher lean-to-fat ratio was associated with a lower risk of self-reported functional limitation after controlling for age, height, pulmonary function impairment, race, education, and smoking history (OR 0.45 per 0.50 increment in lean-to-fat ratio; 95% CI 0.28 to 0.74). Among men, a higher lean-to-fat ratio was associated with a greater distance walked in 6 minutes (mean difference 40 meters per 0.50 ratio increment; 95% CI 9 to 71 meters). In women, the lean-to-fat ratio was associated with an even greater distance walked (mean difference 162 meters per 0.50 increment; 95% CI 97 to 228 meters). In women, higher lean-to-fat ratio was also associated with better Short Physical Performance Battery Scores. In further analysis, the accumulation of greater fat mass, and not the loss of lean mass, was most strongly associated with functional limitation among both sexes.

**Conclusion:**

Body composition is an important non-pulmonary impairment that modulates the risk of functional limitation in COPD, even after taking pulmonary function into account. Body composition abnormalities may represent an important area for screening and preventive intervention in COPD.

## Background

Chronic obstructive pulmonary disease (COPD) is a common chronic health condition, affecting 5–10% of the U.S. population [1,2]. Disability from COPD is substantial, and will likely increase in the U.S. and worldwide [3,4]. Despite these trends, the current understanding of how disability develops in COPD is limited. Although pulmonary function is the most important indicator of physiologic impairment in COPD [5,6], it is a paradoxically a weak predictor of functional limitations [7-9]. Functional limitations, which are decrements in basic physical actions (e.g., mobility, strength), are the key precursors to disability [10,11]. To elucidate the pathway to disability in COPD, we must first understand which physiological impairments, beyond pulmonary function, are important contributors to functional limitation.

An emerging literature suggests that body composition abnormality, especially low body mass index and fat free mass, are an important non-pulmonary physiologic impairment in COPD [12]. In particular, low body mass index or depletion of fat free mass has been associated with increased mortality, lower maximal exercise performance, and poorer health-related quality of life [13-22]. Most of these studies, however, have recruited patients with severe lung disease, oftentimes from pulmonary rehabilitation programs. Consequently, the impact of body composition earlier in the disease, when prevention of functional limitation and disability may still be possible, is less clear. Supporting the possible role of body composition earlier in the disease course, Vestbo and colleagues recently found that low fat free mass and body mass index predicted a higher mortality among patients who had predominately early stage disease [23]. Another study of ambulatory patients with COPD found a relationship between low fat free mass and lower handgrip strength, but there were no differences in dyspnea or health-related quality of life [24]. In the current study, we evaluated the association between body composition and the risk of functional limitation among patients with a broad range of COPD severity recruited from an integrated health care delivery system in Northern California. The goal of this analysis was to study body composition in patients with COPD at a point at which clinical intervention and disability prevention may still be possible.

## Methods

### Overview

The FLOW study of COPD (Function, Living, Outcomes, and Work) is an ongoing prospective cohort study of adult members of a closed panel managed care organization with physician's diagnosis of COPD. Its long-term goal is to determine what factors are responsible for the development of disability in COPD. At baseline assessment, we conducted structured telephone interviews that ascertained COPD status, health status, health-related quality of life, self-reported functional limitations, and sociodemographic characteristics. Subjects then underwent a research clinic visit that included spirometry, bioelectrical impedance, and other physical assessments. Using these baseline data, we evaluated the cross-sectional impact of body composition on the risk of functional limitations among adults with COPD. The study was approved by the University of California, San Francisco Committee on Human Research and the Kaiser Foundation Research Institute's institutional review board.

### Subject recruitment

We studied adult members of Kaiser Permanente (KP), the nation's largest non-profit managed care organization. In Northern California, the Kaiser Permanente Medical Care Program (KPMCP) provides the full spectrum of primary-to-tertiary care to approximately 3.1 million members. In Northern California, KP's share of the regional population ranges from 25 to 30% [25]. The demographic characteristics of KP membership are similar to the overall Northern California population, except for the extremes of income distribution [26].

We identified all adult KPMCP members aged 40–65 years who were recently treated for COPD using a previously described approach [27]. Because an overall study outcome is work disability, younger adults with COPD were recruited. Using KPMCP computerized databases, we identified all subjects who had health care utilization for COPD during the most recent 12 month time period, including 1 or more ambulatory visits, emergency department visits, or hospitalizations with a principal International Classification of Disease (ICD-9) diagnosis code for COPD, which included chronic bronchitis (491), emphysema (492), or COPD (496) PLUS two or more prescriptions for a COPD-related medication during a 12 month window beginning 6 months before the index utilization date and ending 6 months after index date (these medications included inhaled anticholinergic medications, inhaled beta agonists, inhaled corticosteroids, and theophylline). Based on medical record review, we demonstrated that this algorithm is a valid method for identifying adults with COPD [27]. To facilitate attendance at the research clinic, we restricted the sample to persons living within a 30 mile radius of the clinic. The primary care physician for each patient was contacted and given the opportunity to decline contact of their patients. Potential subjects were then contacted by a letter describing the study and given the opportunity to decline by mail. Those not declining were then contacted by telephone to arrange an interview. At the end of the interview, subjects were invited to participate in the research clinic visit. Persons who were found to have other severe life-threatening conditions (e.g., cancer), severe communication or language difficulties (e.g, dementia or stroke), or were not proficient in English were excluded.

This analysis was conducted after the first phase of cohort recruitment. 3144 subjects with COPD were identified and the first randomly sampled 1183 subjects who met all study criteria were eligible for the current analysis. Of the 1183 eligible subjects, 710 (60%) subjects completed structured telephone interviews and 355 (50%) completed the research clinic visit.

### Structured telephone interviews

Each subject underwent a structured telephone interview that was 30–40 minutes in length and conducted using customized computer-assisted telephone interview software. Interviews ascertained age, sex, race-ethnicity, and educational attainment. Cigarette smoking was measured using questions developed for the National Health Interview Survey [28]. As in previous studies, we defined educational attainment as high school or less, some college, or college/graduate degree [4]. Race-ethnicity was categorized as previously described [4].

Self-reported functional limitation was measured using a previously validated approach used by Sternfeld and colleagues, based on questions from the Framingham Disability Study, Established Populations for Epidemiologic Studies of the Elderly, the Nagle scale, and Rosow and Breslau scales [29]. The scale is comprised of 10 questions that assess the degree of difficulty in multiple domains of basic physical functioning such as pushing, stooping, kneeling, getting up from a standing position, lifting lighter or heavier objects, standing, sitting, standing from a seated position, walking up stairs, and walking in the neighborhood. Subjects who indicated "a lot of difficulty" with one or more functions or not doing a function because they were unable or they were told by a doctor not to were defined as having a self-reported functional limitation [29].

### Assessment of body composition and size

Body composition was assessed using bioelectric impedance (BIA).The Quantum II Bioelectrical Body Composition Analyzer (RJL Systems, Clinton Township, MI) was used. While subjects were lying supine, we applied bipolar electrodes on the middle finger of the right hand and the lateral aspect of the right ankle to obtain measures of resistance and reactance. To calculate lean and fat mass, we used established sex-specific regression equations derived from healthy adults living in Northern California who underwent both BIA testing with the Quantum II device and whole-body dual energy x-ray absorptiometry (DEXA) scans [29]. Lean mass and lean-plus-bone mass were derived from these regression equations (in kilograms); fat mass was obtained by subtracting lean-plus-bone mass from weight (because weight = fat mass + lean-plus-bone mass) [29].

A relative measure of body composition, the lean-to-fat ratio, was calculated by dividing lean mass by fat mass. Previous work has established that lean-to-fat ratio is more closely related to functional limitation than lean mass alone. The lean-to-fat ratio was more strongly associated with walking speed and the risk of self-reported functional limitation among elderly adults than were lean or fat mass [29,30]. In addition, the lean-to-fat ratio appeared to mediate the beneficial effects of leisure time physical activity on physical functioning [31]. Lean-to-fat ratio has substantive analytic advantages, because it is independent of body size and is not collinear with height (whereas lean mass and height are collinear).

To assess central adiposity (i.e., visceral fat), we measured sagittal abdominal diameter (SAD). SAD and waist circumference are both excellent measures of visceral fat as determined by MRI or CT scanning [32-36]. SAD appears to be more responsive to weight loss [37]. We chose SAD over waist circumference for this analysis because it correlates more strongly with pulmonary function (both forced vital capacity and forced expiratory volume in 1 second [FEV_1_]) [38]. Moreover, preliminary analysis indicated that SAD was related to overall fat mass, whereas waist circumference was not.

To measure SAD, we used the Holtain Kahn caliper (Holtain Ltd, U.K.). Subjects were studied in the supine position. The examiner located the iliac crests, visualized a line connecting the crests, and marked the center of the abdomen along this line. The caliper was then slid under the back and the caliper's upper arm was slid down until it was 2 cm above the abdominal mark. The caliper was then leveled using the bubble level. The caliper's upper arm was then slid down so that it was just touching, but not compressing, the abdomen. The level position was re-confirmed and the distance in centimeters was determined.

Body mass index, as a more general measure of adiposity, was also determined from height and weight measured at the research clinic visit (weight in kilograms/height in meters^2^). Height was measured by a wall stadiometer in subjects without shoes; weight was measured by a digital scale. Body mass index was categorized into 4 groups using the standard National Heart Lung and Blood Institute/World Health Organization criteria: underweight (< 18.5 kg/m^2^), normal weight 18.5–24.9 kg/m^2^, overweight (25–29.9 kg/m^2^), and obese (≥ 30 kg/m^2^) [39]. Because there were only 9 subjects in the underweight category (3%), they were considered with the normal weight group for analytic purposes.

### Assessment of physical functional limitation

We assessed functional limitations, which are decrements in basic physical actions, using a multifaceted evaluation that combined a survey-based measure (self-reported functional limitation as described above) and physical assessment. Submaximal exercise performance was measured using the Six Minute Walk Test, which was developed by Guyatt and had been widely used in studies of COPD [40,41]. We measured submaximal rather than maximal exercise performance (cardiopulmonary exercise testing) because most daily activities and work tasks are likely to require sustained, submaximal exertion rather than high peak exercise levels. We used a standardized flat, straight course of 30 meters in accordance with American Thoracic Society (ATS) Guidelines [42]. Subjects who use home oxygen or who have a resting oxygen saturation < 90% wore supplemental oxygen. Every 2 minutes, the technician spoke standardized encouragement phrases, as recommended by the ATS guidelines. The primary outcome was the distance walked in 6 minutes.

Lower extremity function was measured using the validated Short Physical Performance Battery [43-45]. The battery included 3 performance measures, which were scored from 0 to 4 points. The standing balance test asks subjects to maintain their feet in a side-by-side, semi-tandem stand (heel of one foot next to the big toe of the other foot), or tandem stand (heel of one foot directly in front of the other foot) for 10 seconds. The maximum score of 4 is assigned for maintaining the tandem stand for 10 seconds; a low score of 1 is assigned for side-by-side standing for 10 seconds, with inability to hold a semi-tandem position for 10 seconds. A test of walking speed requires subjects to walk 4 meters at their normal pace. Participants are assigned a score from 1 to 4 based on the quartile of length of time needed to complete the test. The chair stand test, which reflects lower extremity extensor muscle strength, measures the time required for the subject to stand up and sit down from a chair 5 times with arms folded across the chest. The chair height is standardized for all subjects. Scores from 1 to 4 are assigned based on quartile of length of time to complete the task. A summary performance score integrates the 3 performance measures, ranging from 0 to 12. Previous work indicates that the battery has excellent inter-observer reliability, test-retest reliability, and predictive validity [43-45].

### Pulmonary function assessment

To assess respiratory impairment, we conducted spirometry according to American Thoracic Society (ATS) Guidelines [46,47]. Briefly, subjects were tested in a seated position with a nose clip in place. After the technician demonstrated the procedure, subjects performed at least 3 maximal expiratory maneuvers. If reproducibility criteria are not met (FVC and/or FEV_1 _variability ≤ 0.2 liters), up to 8 maneuvers were obtained. We used the EasyOne™ Frontline spirometer (ndd Medical Technologies, Chelmsford, MA), which meets ATS criteria. To calculate percent predicted pulmonary function values, we used predictive equations derived from NHANES III [48]. Because FEV_1_/FVC ratio is more affected by body size and composition than FEV_1, _we used FEV_1_/FVC in multivariate analysis to control for pulmonary function impairment [38,49]. Based on FEV_1,_FEV_1_/FVC ratio, and respiratory symptoms, COPD severity was staged based on NHLBI/WHO Global Initiative for Chronic Obstructive Lung Disease (GOLD) criteria (stage 0 to IV) [6,50].

### Statistical analysis

Statistical analysis was conducted using SAS software, version 9.1 (SAS Institute, Inc, Cary, NC). We used logistic regression analysis to elucidate the impact of body composition on the risk of self-reported physical functional limitation. The lean-to-fat ratio was chosen as the primary body composition variable (as discussed above). We also examined separate regression models for SAD (an estimate of visceral fat) and BMI (a more general indicator of adiposity). These variables were not included in the same models because of their inter-correlation and the concern for collinearity. To examine potential confounding, 3 sets of analyses are presented that control for covariates: age; age, height, and FEV_1_/FVC; age, height, FEV_1_/FVC, race (white, non-Hispanic vs. other), educational attainment, and smoking history (current smoking and ex-smoking vs. never smoked). To examine the impact of body composition on submaximal exercise performance (Six Minute Walk Test) and lower extremity functioning (Short Physical Performance Battery), multivariate linear regression was used in analogous fashion. Because weight is mostly composed of lean mass and fat mass, it was not included as a covariate in the regression analysis.

A further series of analyses examined the independent impact of lean mass, fat mass, and visceral fat (SAD) on physical functional limitation when considered in the same regression models. Because lean mass was highly correlated with height and fat mass, we used the approach of Sternfeld and colleagues and developed a residual variable for lean mass from its regression on height and fat mass [29]. The residual variable for lean mass (lean mass_resid_) represents the part of lean mass not accounted for by height and fat mass (i.e., the correlations between lean mass_resid _and height, and lean mass_resid _and fat mass are zero). A residual variable was also developed for SAD from its regression on fat mass and lean mass (i.e., SAD_resid _= that part of SAD not accounted for by fat and lean mass).

All regression analyses were stratified by sex, because there was evidence that sex modified the impact of body composition on the risk of functional limitation in many of the analyses. This approach is also consistent with the literature [29,31,51]. To assess the impact of GOLD stage 0 on the results, sensitivity analyses were performed restricted to subjects with GOLD stages I or greater; the results were highly consistent with the primary analysis and are not reported here.

Fat free mass index is sometimes used in studies of body composition. The fat free mass index strongly correlated with lean mass (r = 0.91; p < 0.0001). When key analyses were repeated substituting fat free mass index for lean mass, the results were highly similar to those based on lean mass (data not shown).

## Results

### Subject characteristics

The mean age was 58 (6.2) years and there was a slight predominance of female subjects (60%) (Table 1). The majority of subjects were white (64%), with a substantial proportion of other race-ethnic groups. The majority (82%) indicated smoking during their lifetime. There was a diversity of educational attainment.

**Table 1:** Baseline characteristics of 355 adult patients with COPD in the FLOW cohort study

Characteristic	N (%) or Mean (sd)
Age (years)	58 (6.2)
Sex (female)	212 (60%)
Race (white, non-hispanic)	229 (64%)
Smoking history	
Never smoked	63 (18%)
Current smoker	108 (30%)
Ex-smoker	184 (52%)
Educational attainment	
High school or less	112 (32%)
Some college	151 (43%)
College or graduate degree	92 (26%)

Table 2 shows pulmonary function and body composition measurements. The mean FEV_1 _was 1.71 liters and the majority of subjects were GOLD stage I or greater. A slight majority of subjects were obese (54%) based on BMI. A substantial proportion were overweight (20%) or normal weight (24%), whereas very few were underweight (3%).

**Table 2:** Body composition and pulmonary function among 355 patients with COPD

Measure	Mean (sd) or N (%)
FEV_1 _(liters)	1.71 (0.77)
FEV_1_% predicted (%)	57.9 (22.6)
FEV_1_/FVC	0.60 (0.16)
GOLD Stage	
0	106 (30%)
1	18 (5%)
2	96 (27%)
3	73 (21%)
4	62 (17%)
Height (meters)	1.67 (0.092)
Weight (kg)	86.8 (24.1)
Lean body mass (kg)	49.3 (12.6)
Fat body mass (kg)	34.6 (16.6)
Sagittal abdominal diameter (cm)	24.7 (5.0)
BMI	
Underweight (< 18.5 kg/m^2^)	9 (3%)
Normal weight (18.5–24.9 kg/m^2^)	85 (24%)
Overweight (25.0–29.9 kg/m^2^)	70 (20%)
Obese (≥ 30 kg/m^2^)	191 (54%)

### Body composition and functional limitation in COPD

In men, a higher sagittal abdominal diameter was associated with a greater risk of self-reported functional limitation, but the confidence interval was wide and did not exclude no effect (OR 1.09 per 1 cm increment; 95% CI 0.99 to 1.21) (Table 3). There was no apparent relation between lean-fat ratio or BMI and self-reported functional limitation.

**Table 3:** Body composition and the risk of self-reported functional limitation among 355 patients with COPD

Measure of body composition	Age adjusted	Age, height, FEV_1_/FVC adjusted	Age, height, FEV_1_/FVC, race, education, and smoking adjusted
	OR (95% CI)	OR (95% CI)	OR (95% CI)
**MEN (n = 143)**			
Lean/fat ratio	1.02 (0.87 to 1.20)	0.98 (0.82 to 1.16)	0.99 (0.83 to 1.18)
SAD	1.07 (0.99 to 1316)	1.10 (1.0 to 1.20)	1.09 (0.99 to 1.21)
BMI			
Normal weight	Referent	Referent	Referent
Overweight	0.36 (0.10 to 1.27)	0.42 (0.11 to 1.56)	0.46 (0.12 to 1.81)
Obese	0.92 (0.38 to 2.24)	1.16 (0.44 to 3.03)	1.12 (0.39 to 3.20)
**WOMEN (n = 212)**			
Lean/fat ratio	**0.49 (0.32 to 0.75)**	**0.44 (0.27 to 0.70)**	**0.45 (0.28 to 0.74)**
SAD	**1.13 (1.06 to 1.21)**	**1.15 (1.07 to 1.24)**	**1.15 (1.07 to 1.23)**
BMI			
Normal weight	Referent	Referent	Referent
Overweight	0.79 (0.28 to 2.24)	0.82 (0.28 to 2.37)	0.74 (0.25 to 2.18)
Obese	**3.0 (1.45 to 6.23)**	**3.77 (1.70 to 8.37)**	**3.50 (1.53 to 8.01)**

Results are from separate multivariate logistic regression of self-reported functional limitation regressed on body composition measures plus covariates.

Lean/fat ratio = derived from bioelectrical impedance. Odds ratios are expressed per 0.50 increment in the ratio.

SAD = sagittal abdominal diameter, an estimate of visceral fat. Odds ratios are expressed per 1 cm increment.

BMI = body mass index, an estimate of adiposity; normal weight (18.5 to 24.9 kg/m^2^) overweight = 25.0 to 29.9 kg/m^2^, obese = 30.0 kg/m^2 ^or greater; only 9/355 (2.5%) of subjects were in underweight category (< 18.5 kg/m^2^) so these were included in the normal weight group.

Among women, a higher lean-to-fat ratio was associated with a lower risk of self-reported functional limitation in the fully adjusted model (OR 0.45 per 0.50 increment in lean-to-fat ratio; 95% CI 0.28 to 0.74). Higher sagittal abdominal diameter and obese body mass index were also related to a greater risk of functional limitation (OR 1.15 per 1 cm increment; 95% CI 1.07 to 1.23 and OR 3.50 for obese vs. normal BMI; 95% CI 1.53 to 8.01, respectively).

Body composition was associated with exercise performance on the Six Minute Walk Test, although the effects were greater for woman than for men (Table 4). Among men, a higher lean-to-fat ratio was associated with a greater distance walked in 6 minutes in the fully adjusted analysis (mean difference 40 meters per 0.50 ratio increment; 95% CI 9 to 71 meters). In women, the lean-to-fat ratio was associated with an even greater distance walked (mean difference 162 meters per 0.50 increment; 95% CI 97 to 228 meters). Larger sagittal abdominal diameter and obese BMI were also related to less distance walked in 6 minutes in both sexes (Table 4).

**Table 4:** Body composition and exercise performance on the Six Minute Walk Test among patients with COPD

Measure of body composition	Age adjusted	Age, height, FEV_1_/FVC adjusted	Age, height, FEV_1_/FVC, race, education, and smoking adjusted
	Mean meters (95% CI)	Mean meters (95% CI)	Mean meters (95% CI)
**MEN (n = 143)**			
Lean/fat ratio	**39 (9 to 69)**	**42 (11 to 72)**	**40 (9 to 71)**
SAD	**-29 (-42 to -15)**	**-34 (-47 to -20)**	**-34 (-48 to -19)**
BMI			
Normal weight	Referent	Referent	Referent
Overweight	27 (-171 to 225)	-28 (-225 to 169)	-88 (-295 to 120)
Obese	**-264 (-431 to -97)**	**-263 (-431 to -95)**	**-269 (-451 to -87)**
**WOMEN (n = 212)**			
Lean/fat ratio	**140 (82 to 199)**	**159 (96 to 223)**	**162 (97 to 228)**
SAD	**-34 (-43 to -24)**	**-38 (-48 to -28)**	**-39 (-49 to -28)**
BMI			
Normal weight	Referent	Referent	Referent
Overweight	-42 (-202 to 117)	-56 (-217 to 106)	-49 (-214 to 115)
Obese	**-340 (-462 to -218)**	**-392 (-521 to -262)**	**-398 (-531 to -264)**

Results are from separate multivariate linear regression of distance in meters walked in 6 minutes regressed on body composition measures plus covariates.

Lean/fat ratio = derived from bioelectrical impedance. Results are expressed per 0.50 increment in the ratio.

SAD = sagittal abdominal diameter, an estimate of visceral fat. Results are expressed per 1 cm increment.

BMI = body mass index, an estimate of adiposity; normal weight (18.5 to 24.9 kg/m^2^) overweight = 25.0 to 29.9 kg/m^2^, obese = 30.0 kg/m^2 ^or greater; only 9/355 (2.5%) of subjects were in underweight category (< 18.5 kg/m^2^) so these were included in the normal weight group

Among men, higher sagittal abdominal diameter was associated with worse performance on the walking speed score and summary performance score of the Short Physical Performance Battery (Table 5). In the female stratum, lean-to-fat ratio, sagittal abdominal diameter, and obese BMI were all related to walking speed score, chair stand scores, and summary performance scores in the expected directions.

**Table 5:** The influence of body composition on physical performance among patients with COPD

Measure of body composition	Standing balance score	Walking speed score	Chair stand score	**Summary performance score**
**MEN (n = 143)**				
Lean/fat ratio	0.007 (-0.034 to 0.049)	0.020 (-0.021 to 0.060)	0.038 (-0.05 to 0.13)	0.65 (-0.070 to 0.20)
SAD	-0.015 (-0.035 to 0.006)	-0.019 (-0.039 to 0.0015)*	-0.033 (-0.076 to 0.011)	**-0.067 (-0.13 to 0.00)**
BMI				
Normal weight	Referent	Referent	Referent	Referent
Overweight	0.060 (-0.22 to 0.34)	-0.081 (-0.36 to 0.20)	0.45 (-0.14 to 1.04)	0.43 (-0.48 to 1.35)
Obese	-0.13 (-0.38 to 0.11)	-0.14 (-0.38 to 0.11)	-0.008 (-0.053 to 0.51)	-0.28 (-1.08 to 0.52)
**WOMEN (n = 212)**				
Lean/fat ratio	0.070 (-0.023 to 0.16)	0.10 (-0.007 to 0.43)*	**0.35 (0.17 to 1.05)**	**0.52 (0.24 to 0.80)**
SAD	-0.011(-0.028 to 0.006)	**-0.033 (-0.052 to -0.14)**	**-0.053 (-0.085 to -0.021**)	**-0.097 (-0.015 to -0.047)**
BMI				
Normal weight	Referent	Referent	Referent	Referent
Overweight	-0.003 (-0.25 to 0.24)	-0.016 (-0.30 to 0.27)	-0.082 (-0.55 to 0.39)	-0.10 (-0.84 to 0.64)
Obese	-0.029 (-0.23 to 0.17)	**-0.38 (-0.61 to -0.15)**	**-0.60 (-0.98 to -0.22)**	**-1.00 (-1.61 to -0.40)**

All results are mean score (95% CI) from multivariate linear regression controlling for age, height, FEV1/FVC, race, education, and smoking history.

Results are from separate multivariate linear regression of each score regressed on body composition measures plus covariates.

Boldface when p < 0.05 *p = 0.07

Each Short Physical Performance subscale score ranges from 0–4, with higher scores reflecting more favorable performance. Summary performance score is sum of each subscale score and ranges from 0–12.

Lean/fat ratio = derived from bioelectrical impedance. Results are expressed per 0.50 increment in the ratio.

SAD = sagittal abdominal diameter, an estimate of visceral fat. Results are expressed per 1 cm increment.

BMI = body mass index, an estimate of adiposity; normal weight (18.5 to 24.9 kg/m^2^) overweight = 25.0 to 29.9 kg/m^2^, obese = 30.0 kg/m^2 ^or greater; only 9/355 (2.5%) of subjects were in underweight category (< 18.5 kg/m^2^) so these were included in the normal weight group

### Relative contribution of lean mass, fat mass, and visceral fat to functional limitation

To further elucidate the impact of body composition on functional limitation, lean mass (residual), fat mass, and visceral fat (estimated by sagittal abdominal diameter residual) were included simultaneously in each fully adjusted multivariate model (see Methods). Among men, higher fat mass was associated with a decrement in the Six Minute Walk Test (-13 meters per 1 kg fat mass increment; 95% CI -21 to -5 meters) and was possibly related to a greater risk of self-reported functional limitations (OR 1.06 per 1 kg increment; 95% CI 0.99 to 1.13) (Table 6). Higher visceral fat, as estimated by sagittal abdominal diameter, was also related to poorer walk performance (-38 meters per 1 cm increment; 95% CI -68 to -7 meters).

**Table 6:** Independent influence of lean and fat mass on functional limitation in COPD

Measure of body composition	Self-reported functional limitation	Six Minute Walk Test	SPPB Summary Performance Score
	OR (95% CI)	Mean (95% CI)	Mean (95% CI)
**MEN (n = 143)**			
Lean mass_resid_	1.0 (0.89 to 1.12)	5 (-10 to 20)	0.018 (-0.05 to 0.09)
Fat mass	1.06 (0.99 to 1.13)*	**-13 (-21 to -5)**	-0.025 (-0.062 to 0.013)
SAD_resid_	0.95 (0.76 to 1.19)	**-38 (-68 to -7)**	-0.097 (-0.24 to 0.043)
**WOMEN (n = 212)**			
Lean mass_resid_	1.005 (0.91 to 1.12)	-14 (-31 to 3)	0.034 (-0.044 to 0.11)
Fat mass	**1.04 (1.017 to 1.067)**	**-11 (-15 to -8)**	**-0.037 (-0.053 to -0.020)**
SAD_resid_	1.09 (0.94 to 1.26)	-16 (-39 to 7)	-0.012 (-0.12 to 0.097)

Logistic or linear multivariate regression including variables shown plus age, FEV_1_/FVC, height, race, education, and smoking. *p = 0.077

Results are for 1 kg increment in lean or fat mass OR per 1 cm increment in SAD

Lean mass_resid _= residual variable for lean mass removing the contribution of fat mass and height;

SAD_resid _= residual variable for sagittal abdominal diameter removing the contribution of lean mass and fat mass (see Methods)

Among women, higher fat mass was related to a greater risk of functional limitations (OR 1.04 per 1 kg increment in fat mass; 95% CI 1.017 to 1.067), 11 meter decrement in the distance walked in six minutes (95% CI -15 to -8), and a poorer SPPB summary performance score (-0.037 points per 1 kg increment; 95% CI -0.053 to -0.020).

## Discussion

Body composition abnormality was associated with an increased risk of functional limitation among patients with COPD who had a wide spectrum of severity, especially among women. A lean-to-fat mass ratio was associated with a decreased risk of self-reported functional limitation, better submaximal exercise performance (Six Minute Walk Test), and better lower extremity functioning (Short Physical Performance Battery), even after controlling for pulmonary function impairment and other covariates. Higher measures of total adiposity (BMI) and central adiposity (SAD) were also related to greater functional limitation among women. In men, the salutary effect of lean-to-fat ratio was absent for self-reported functional limitations and lower extremity functioning; it had a beneficial, albeit attenuated, impact on submaximal exercise performance. In further analysis, the accumulation of greater fat mass, and not the loss of lean mass, was most strongly associated with functional limitation among both sexes. In sum, body composition is an important non-pulmonary impairment that modulates the risk of functional limitation in COPD, even after taking pulmonary function into account.

Although low fat free mass has been linked with mortality in COPD, less is known about its impact on functional limitation, which is a more proximal outcome [16,23]. Depletion of fat free mass has been linked with poorer submaximal exercise performance and health related quality of life among patients with very advanced disease who were participating in pulmonary rehabilitation programs [15,20]. A more recent study of ambulatory patients with moderate COPD severity, however, found no relation between fat free mass and dyspnea or health-related quality of life, but walking and other related functional limitations were not evaluated [24]. Our study demonstrated that body composition has an important impact on functional limitation among persons with earlier stage disease, when prevention may still be possible. Compared to earlier studies, we were also able to parse out the independent effects of lean mass and fat mass.

Our results suggest that COPD may accelerate the impact of body composition that occurs with normal ageing. Among elderly adults who were an average of 11 years older than our cohort, the lean-to-fat ratio was an important determinant of functional limitation, especially among women [29-31]. Greater fat mass was the most important predictor of more functional limitation; lean mass was only predictive in relation to fat mass. Other population-based studies of the elderly have also suggested that fat mass is the most important influence on functional limitation [52-54]. Overall, it appears that the increase of fat mass, and not simply the loss of lean mass, is an important precursor for the development of functional limitation and that this process is occurring at an earlier age in COPD than in the general population. This differs from the traditional view, which posits that lean mass depletion is the most important determinant in COPD [55].

The present study is subject to several limitations. Although the inclusion criteria require health care utilization for COPD, misclassification of asthma could affect the study results. Our COPD definition required concomitant treatment with COPD medications to increase the specificity of the definition. The observed lifetime smoking prevalence was similar to that in other population-based epidemiologic studies of COPD, supporting the diagnosis of COPD over asthma [1,56]. We also previously demonstrated the validity of our approach using medical record review [27]. Moreover, we demonstrated that all patients met the GOLD criteria for COPD. Nonetheless, we cannot exclude the possibility that some subjects, especially GOLD stage 0, have conditions other than COPD. For the present analysis, we would expect such misclassification to have a conservative effect (i.e., reducing the impact of body composition on functional limitation).

Because our focus was on disability prevention, we intentionally sampled younger adults with COPD. Therefore, these results may underestimate the impact of body composition among older patients with COPD In addition, Kaiser Permanente members, because they have health care access, may also be different than the general population of adults with COPD. Mitigating these limitations, the sociodemographic characteristics of Northern California Kaiser Permanente members are similar to those of the regional population, with some under-representation of income extremes [25,26]. Moreover, selection bias could have been introduced by non-participation in the study, but the demographic characteristics of those who did and did not participate are similar (data not shown). Our subjects also had a low prevalence of underweight and a high prevalence of obesity, which likely reflects the broad range of disease severity; this could reduce generalizability to populations of end-stage COPD patients who often have more underweight persons.

We did not perform DEXA in this cohort, which is the best clinically available measure of lean and fat mass. We did, however, use regression equations to estimate fat and lean mass that were recently developed and validated for subjects living within the catchment area of the study [29]. There are, however, alternative equations for estimating body composition [15]. Another limitation was inadequate statistical power to evaluate the impact of lean and fat mass within BMI categories. In addition, we did not have a control group for this analysis so the relative impact of body composition on patients with COPD compared to the general population could not be evaluated.

## Conclusion

Pulmonary function impairment, although it is the most salient abnormality in COPD, cannot explain why some patients develop functional limitations and disability and others do not. A lower lean-to-fat ratio is associated with greater functional limitation, especially among women. Moreover, higher fat mass has a particularly negative impact on function. Consequently, body composition abnormalities may represent an important area for screening and preventive intervention in COPD. Further studies are needed to evaluate the efficacy of these interventions.

## Competing interests

The author(s) declare that they have no competing interests.

## Authors' contributions

ME designed the study, analyzed the data, and wrote the paper; PB assisted with study design and writing; SS assisted with study design and implementation; EY assisted with writing and reviewing of the final manuscript;PL managed study recruitment and subject examination and assisted with writing the manuscript;IT assisted with the analysis; LA assisted with the analysis and writing; CI assisted with study implementation and writing of the paper.

## Appendix

Assessment of self-reported functional limitations

The next questions ask about difficulties that you might have with common activities. For the next items, please tell me what level of difficulty you have had during the past month: a lot of difficulty, some difficulty, a little difficulty, or no difficulty.

During the past month, how much difficulty have you had....

In pushing objects, like a living room chair?

In stooping, crouching, or kneeling?

In getting up from a stooping, crouching, or kneeling position?

In lifting or carrying items under 10 pounds, like a bag of potatoes?

In lifting or carrying items over 10 pounds, like a bag of groceries?

In standing in place for 15 minutes or longer?

In sitting for long periods, say 1 hour?

In standing up after sitting in a chair?

In walking alone up and down a flight of stairs?

In walking two or three neighborhood blocks?

## References

[B1] Mannino DM, Homa DM, Akinbami LJ, Ford ES, Redd SC (2002). Chronic obstructive pulmonary disease surveillance – United States, 1971–2000. MMWR Surveill Summ.

[B2] Halbert RJ, Isonaka S, George D, Iqbal A (2003). Interpreting COPD prevalence estimates: what is the true burden of disease?. Chest.

[B3] Murray CJ, Lopez AD (1997). Alternative projections of mortality and disability by cause 1990–2020: Global Burden of Disease Study. Lancet.

[B4] Eisner MD, Yelin EH, Trupin L, Blanc PD (2002). The Influence of Chronic Respiratory Conditions on Health Status and Work Disability. Am J Public Health.

[B5] Mannino DM (2002). COPD: epidemiology, prevalence, morbidity and mortality, and disease heterogeneity. Chest.

[B6] Pauwels RA, Buist AS, Calverley PM, Jenkins CR, Hurd SS (2001). Global strategy for the diagnosis, management, and prevention of chronic obstructive pulmonary disease. NHLBI/WHO Global Initiative for Chronic Obstructive Lung Disease (GOLD) Workshop summary. Am J Respir Crit Care Med.

[B7] Bestall JC, Paul EA, Garrod R, Garnham R, Jones PW, Wedzicha JA (1999). Usefulness of the Medical Research Council (MRC) dyspnoea scale as a measure of disability in patients with chronic obstructive pulmonary disease. Thorax.

[B8] Jette DU, Manago D, Medved E, Nickerson A, Warzycha T, Bourgeois MC (1997). The disablement process in patients with pulmonary disease. Phys Ther.

[B9] Williams SJ, Bury MR (1989). 'Breathtaking': the consequences of chronic respiratory disorder. Int Disabil Stud.

[B10] Verbrugge LM, Jette AM (1994). The disablement process. Soc Sci Med.

[B11] Verbrugge LM, Juarez L (2001). Profile of arthritis disability. Public Health Rep.

[B12] Andreassen H, Vestbo J (2003). Chronic obstructive pulmonary disease as a systemic disease: an epidemiological perspective. Eur Respir J Suppl.

[B13] Landbo C, Prescott E, Lange P, Vestbo J, Almdal TP (1999). Prognostic value of nutritional status in chronic obstructive pulmonary disease. Am J Respir Crit Care Med.

[B14] Prescott E, Almdal T, Mikkelsen KL, Tofteng CL, Vestbo J, Lange P (2002). Prognostic value of weight change in chronic obstructive pulmonary disease: results from the Copenhagen City Heart Study. Eur Respir J.

[B15] Schols AM, Mostert R, Soeters PB, Wouters EF (1991). Body composition and exercise performance in patients with chronic obstructive pulmonary disease. Thorax.

[B16] Slinde F, Gronberg A, Engstrom CP, Rossander-Hulthen L, Larsson S (2005). Body composition by bioelectrical impedance predicts mortality in chronic obstructive pulmonary disease patients. Respir Med.

[B17] Baarends EM, Schols AM, Mostert R, Wouters EF (1997). Peak exercise response in relation to tissue depletion in patients with chronic obstructive pulmonary disease. Eur Respir J.

[B18] Celli BR, Cote CG, Marin JM, Casanova C, Montes de Oca M, Mendez RA, Pinto Plata V, Cabral HJ (2004). The body-mass index, airflow obstruction, dyspnea, and exercise capacity index in chronic obstructive pulmonary disease. N Engl J Med.

[B19] Gray-Donald K, Gibbons L, Shapiro SH, Macklem PT, Martin JG (1996). Nutritional status and mortality in chronic obstructive pulmonary disease. Am J Respir Crit Care Med.

[B20] Mostert R, Goris A, Weling-Scheepers C, Wouters EF, Schols AM (2000). Tissue depletion and health related quality of life in patients with chronic obstructive pulmonary disease. Respir Med.

[B21] Yoshikawa M, Yoneda T, Kobayashi A, Fu A, Takenaka H, Narita N, Nezu K (1999). Body composition analysis by dual energy X-ray absorptiometry and exercise performance in underweight patients with COPD. Chest.

[B22] Yoshikawa M, Yoneda T, Takenaka H, Fukuoka A, Okamoto Y, Narita N, Nezu K (2001). Distribution of muscle mass and maximal exercise performance in patients with COPD. Chest.

[B23] Vestbo J, Prescott E, Almdal T, Dahl M, Nordestgaard BG, Andersen T, Sorensen TI, Lange P (2006). Body mass, fat-free body mass, and prognosis in patients with chronic obstructive pulmonary disease from a random population sample: findings from the copenhagen city heart study. Am J Respir Crit Care Med.

[B24] Vermeeren MA, Creutzberg EC, Schols AM, Postma DS, Pieters WR, Roldaan AC, Wouters EF (2006). Prevalence of nutritional depletion in a large out-patient population of patients with COPD. Respir Med.

[B25] Karter AJ, Ferrara A, Liu JY, Moffet HH, Ackerson LM, Selby JV (2002). Ethnic disparities in diabetic complications in an insured population. JAMA.

[B26] Krieger N (1992). Overcoming the absence of socioeconomic data in medical records: validation and application of a census-based methodology. American Journal of Public Health.

[B27] Sidney S, Sorel M, Quesenberry CP, DeLuise C, Lanes S, Eisner MD (2005). COPD and incident cardiovascular disease hospitalizations and mortality: Kaiser Permanente Medical Care Program. Chest.

[B28] (1999). Cigarette smoking among adults – United States, 1997. Mmwr Morbidity and Mortality Weekly Report.

[B29] Sternfeld B, Ngo L, Satariano WA, Tager IB (2002). Associations of body composition with physical performance and self-reported functional limitation in elderly men and women. Am J Epidemiol.

[B30] Tager IB, Haight T, Sternfeld B, Yu Z, van Der Laan M (2004). Effects of physical activity and body composition on functional limitation in the elderly: application of the marginal structural model. Epidemiology.

[B31] Haight T, Tager I, Sternfeld B, Satariano W, van der Laan M (2005). Effects of body composition and leisure-time physical activity on transitions in physical functioning in the elderly. Am J Epidemiol.

[B32] Clasey JL, Bouchard C, Teates CD, Riblett JE, Thorner MO, Hartman ML, Weltman A (1999). The use of anthropometric and dual-energy X-ray absorptiometry (DXA) measures to estimate total abdominal and abdominal visceral fat in men and women. Obes Res.

[B33] Hill JO, Sidney S, Lewis CE, Tolan K, Scherzinger AL, Stamm ER (1999). Racial differences in amounts of visceral adipose tissue in young adults: the CARDIA (Coronary Artery Risk Development in Young Adults) study. Am J Clin Nutr.

[B34] Pouliot MC, Despres JP, Lemieux S, Moorjani S, Bouchard C, Tremblay A, Nadeau A, Lupien PJ (1994). Waist circumference and abdominal sagittal diameter: best simple anthropometric indexes of abdominal visceral adipose tissue accumulation and related cardiovascular risk in men and women. Am J Cardiol.

[B35] Kvist H, Chowdhury B, Grangard U, Tylen U, Sjostrom L (1988). Total and visceral adipose-tissue volumes derived from measurements with computed tomography in adult men and women: predictive equations. Am J Clin Nutr.

[B36] Sjostrom L (1991). A computer-tomography based multicompartment body composition technique and anthropometric predictions of lean body mass, total and subcutaneous adipose tissue. Int J Obes.

[B37] van der Kooy K, Leenen R, Seidell JC, Deurenberg P, Visser M (1993). Abdominal diameters as indicators of visceral fat: comparison between magnetic resonance imaging and anthropometry. Br J Nutr.

[B38] Santana H, Zoico E, Turcato E, Tosoni P, Bissoli L, Olivieri M, Bosello O, Zamboni M (2001). Relation between body composition, fat distribution, and lung function in elderly men. Am J Clin Nutr.

[B39] Flegal KM (2005). Epidemiologic aspects of overweight and obesity in the United States. Physiol Behav.

[B40] Guyatt GH, Sullivan MJ, Thompson PJ, Fallen EL, Pugsley SO, Taylor DW, Berman LB (1985). The 6-minute walk: a new measure of exercise capacity in patients with chronic heart failure. Can Med Assoc J.

[B41] Sciurba F, Criner GJ, Lee SM, Mohsenifar Z, Shade D, Slivka W, Wise RA (2003). Six-minute walk distance in chronic obstructive pulmonary disease: reproducibility and effect of walking course layout and length. Am J Respir Crit Care Med.

[B42] (2002). ATS statement: guidelines for the six-minute walk test. Am J Respir Crit Care Med.

[B43] Guralnik JM, Ferrucci L, Simonsick EM, Salive ME, Wallace RB (1995). Lower-extremity function in persons over the age of 70 years as a predictor of subsequent disability. N Engl J Med.

[B44] Guralnik JM, Ferrucci L, Pieper CF, Leveille SG, Markides KS, Ostir GV, Studenski S, Berkman LF, Wallace RB (2000). Lower extremity function and subsequent disability: consistency across studies, predictive models, and value of gait speed alone compared with the short physical performance battery. J Gerontol A Biol Sci Med Sci.

[B45] Guralnik JM, Simonsick EM, Ferrucci L, Glynn RJ, Berkman LF, Blazer DG, Scherr PA, Wallace RB (1994). A short physical performance battery assessing lower extremity function: association with self-reported disability and prediction of mortality and nursing home admission. J Gerontol.

[B46] American Thoracic Society (1987). Standardization of spirometry – 1987 update. Statement of the American Thoracic Society. American Review of Respiratory Disease.

[B47] (1995). Standardization of Spirometry, 1994 Update. American Thoracic Society. Am J Respir Crit Care Med.

[B48] Hankinson JL, Odencrantz JR, Fedan KB (1999). Spirometric reference values from a sample of the general U.S. population. Am J Respir Crit Care Med.

[B49] Lazarus R, Sparrow D, Weiss ST (1997). Effects of obesity and fat distribution on ventilatory function: the normative aging study. Chest.

[B50] Fabbri LM, Hurd SS (2003). Global Strategy for the Diagnosis, Management and Prevention of COPD: 2003 update. Eur Respir J.

[B51] Tager IB, Haight TJ, Hollenberg M, Satariano WA (2003). Physical functioning and mortality in older women: an assessment of energy costs and level of difficulty. J Clin Epidemiol.

[B52] Davison KK, Ford ES, Cogswell ME, Dietz WH (2002). Percentage of body fat and body mass index are associated with mobility limitations in people aged 70 and older from NHANES III. J Am Geriatr Soc.

[B53] Zamboni M, Turcato E, Santana H, Maggi S, Harris TB, Pietrobelli A, Heymsfield SB, Micciolo R, Bosello O (1999). The relationship between body composition and physical performance in older women. J Am Geriatr Soc.

[B54] Visser M, Langlois J, Guralnik JM, Cauley JA, Kronmal RA, Robbins J, Williamson JD, Harris TB (1998). High body fatness, but not low fat-free mass, predicts disability in older men and women: the Cardiovascular Health Study. Am J Clin Nutr.

[B55] Wouters EF (2006). Muscle wasting in chronic obstructive pulmonary disease: to bother and to measure!. Am J Respir Crit Care Med.

[B56] Eisner MD, Balmes J, Katz PP, Trupin L, Yelin EH, Blanc PD (2005). Lifetime environmental tobacco smoke exposure and the risk of chronic obstructive pulmonary disease. Environ Health.

